# Vorhersage und frühzeitige Identifikation einer postpartalen Depression: Ergebnisse der longitudinalen RiPoD-Studie im Kontext der Literatur

**DOI:** 10.1007/s00115-024-01726-1

**Published:** 2024-08-26

**Authors:** Susanne Nehls, Juergen Dukart, Christian Enzensberger, Elmar Stickeler, Simon B. Eickhoff, Natalia Chechko

**Affiliations:** 1 Klinik für Psychiatrie, Psychotherapie and Psychosomatik, Uniklinik RWTH Aachen, Deutschland; 2 Institute of Neuroscience and Medicine, JARA-Institute Brain Structure Function Relationship (INM-10), Forschungszentrum Jülich, Jülich, Deutschland; 3 Institute of Neuroscience and Medicine, Brain & Behavior (INM-7), Forschungszentrum Jülich, Jülich, Deutschland; 4 Institute of Systems Neuroscience, Medizinische Fakultät, Heinrich-Heine-Universität Düsseldorf, Düsseldorf, Deutschland; 5 Klinik für Gynäkologie und Geburtshilfe, Uniklinik RWTH Aachen, Aachen, Deutschland; 6 Klinik für Psychiatrie, Psychotherapie and Psychosomatik, Uniklinik RWTH Aachen, Pauwelsstraße 23, 52070 Aachen, Deutschland

**Keywords:** Postpartum, Depression, Longitudinal, Hirnbildgebung, Vorhersage, Postpartum, Depression, Longitudinal, Brain imaging, Prediction

## Abstract

Die ersten 4 bis 6 Wochen nach der Geburt werden als Zeitfenster für den Beginn der postpartalen Depression (PPD) definiert. Trotz dieses bekannten Zeitfensters gibt es gravierende Lücken in der Identifizierung und Behandlung der PPD. In der Studie „Risk for Postpartum Depression“ (RiPoD) untersuchten wir spezifische Risikofaktoren und Prädiktoren für postpartale psychische Anpassungsprozesse und stellen die Ergebnisse dieser Studie im Rahmen einer Übersichtsarbeit zum internationalen Forschungsstand vor. Die dynamischen neuroplastischen Veränderungen des mütterlichen Gehirns in der Schwangerschaft und im Wochenbett scheinen in enger Verbindung mit peripartal fluktuierenden Hormonspiegeln zu stehen und diese könnten gemeinsam die Entwicklung postpartaler Stimmungsphänomene beeinflussen. Zu den relevanten Einflussgrößen der PPD gehören hormonelle Risikofaktoren, wie der Babyblues und das prämenstruelle Syndrom. Die Kombination beider Faktoren ermöglicht es, das individuelle PPD-Risiko mit einer Sensitivität von 83 % in der ersten Woche postpartum vorherzusagen. Eine anschließende digitale Überwachung der Symptomentwicklung in den ersten 6 Wochen postpartal erlaubte eine präzise Identifikation von Frauen mit PPD. Das Verständnis der Interaktion von hormonellen Schwankungen, Neuroplastizität und psychiatrischen Störungen bietet einen wichtigen Ansatzpunkt für zukünftige Forschungen. Die frühzeitige Identifikation und Diagnose der PPD sowie kritischer Risikofaktoren lassen sich leicht in die klinische Routine und den Alltag der Patientinnen integrieren, wodurch Frauen mit hohem Risiko für eine gezielte Überwachung identifiziert werden können.

## Abstract (en)

The first 4–6 weeks after childbirth are defined as the onset time for postpartum depression (PPD). Despite this known time frame there are significant gaps in the identification and treatment of PPD. The risk for postpartum depression (RiPoD) study investigated specific risk factors and predictors of postpartum psychological adjustment processes and the results are presented within the framework of a state of the art review of research. The dynamic neuroplastic changes in the maternal brain during pregnancy and the postpartum period appear to be closely linked to peripartum hormone fluctuations, which jointly influence the development of postpartum mood disorders. Hormonal risk factors such as baby blues and premenstrual syndrome have been found to have a bearing on PPD. The combination of these two factors predicts the risk of PPD with 83% sensitivity within the first week postpartum. Follow-up digital monitoring of symptom development in the first 6 weeks postpartum has enabled an accurate identification of women with PPD. Understanding the interaction between hormone fluctuations, neuroplasticity and psychiatric disorders should be an important target for future research. Early identification and diagnosis of PPD can be easily integrated into the clinical routine and everyday life.

## Hintergrund

Das Diagnostische und Statistische Manual Psychischer Störungen (DSM‑5; [1]) und die International Statistical Classification of Diseases and Related Health Problems (ICD-11; [55]) definieren die ersten 4 bis 6 Wochen nach Kindsgeburt – die subakute postpartale Phase – als den Beginn postpartaler psychischer Erkrankungen, wobei die postpartale Depression (PPD) die Häufigste ist [31]. Wie die Major-Depression (MDD) hat die PPD multifaktorielle Ursachen, zu denen genetische Prädisposition, psychosoziale Stressoren, stressige und traumatische Lebensereignisse sowie frühere depressive Episoden gehören [31, 41]. Darüber hinaus gibt es bei der PPD spezifische Faktoren, die eng mit Schwangerschaft und Geburt verbunden sind, wodurch sich die PPD von der MDD unterscheidet [41].

Trotz der Bekanntheit des kritischen Zeitfensters der ersten 4 bis 6 Wochen nach der Geburt gibt es weitreichende Mängel bei der Untersuchung, Erkennung und Behandlung der PPD [1, 44]. Um diese Forschungs- und Behandlungslücken zu schließen, wurde die Risk-of-Postpartum-Depression(RiPoD)-Studie durchgeführt, in der insgesamt 938 euthyme Frauen 1 bis 6 Tage nach der Geburt rekrutiert wurden. Neben der 12-wöchigen digital basierten Beobachtung der Entwicklung von Stimmung und Stress wurden auch hormonelle und neuroplastische Anpassungsprozesse in den ersten postpartalen Wochen sowie spezifische Risikofaktoren und Prädiktoren für postpartale psychische Anpassungsprozesse untersucht und zueinander in Beziehung gesetzt . Im Folgenden sollen im Rahmen einer Übersichtsarbeit die Ergebnisse der RiPoD-Studie im Kontext des internationalen Standes der Forschung vorgestellt werden.

**Abb. 1:**
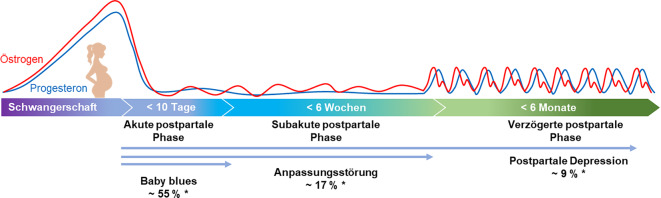
Die drei Phasen des Wochenbettes unterteilt in akute, subakute und verzögerte postpartale Phase, unter Berücksichtigung der hormonellen Verläufe sowie der Zeitfenster der postpartalen Störungen (Babyblues, Anpassungsstörung, postpartale Depression). *Sternchen* Prävalenzzahlen sind entnommen aus Chechko et al. [11] und Hahn et al. [22]

## Hormonelle und neuroplastische Anpassungsprozesse in den ersten postpartalen Wochen und deren Zusammenhang mit den affektiven Zuständen junger Mütter

Während der Schwangerschaft sorgt der kontinuierliche und starke Anstieg der zirkulierenden Sexualhormone, insbesondere Progesteron und Östradiol [18, 27] für die Aufrechterhaltung der Schwangerschaft und fördert die Veränderungen des weiblichen Körpers [26]. Es wird angenommen, dass die fluktuierenden Hormone durch das Überwinden der Blut-Hirn-Schranke das mütterliche Gehirn verändern [18, 44]. Tatsächlich zeigt die Forschung der letzten Jahrzehnte einen schwangerschaftsbedingten Verlust an grauer Substanz („gray matter volume“, GMV) und kortikaler Dicke, der im 2. und 3. Trimester sowie bei der Geburt im Vergleich zur Zeit vor der Schwangerschaft zu beobachten ist [24, 35, 39]. Zusätzlich scheinen Estradiolwerte des 3. Trimesters GMV-Veränderungen widerzuspiegeln, die von der Zeit vor der Schwangerschaft bis zur Geburt auftreten, was zum Gesamtmuster der beobachteten neuronalen Veränderungen beiträgt [24]. Die ersten 6–12 h bis zu den ersten 10 Tagen nach der Geburt – die akute postpartale Phase – sind durch eine plötzliche Depletion der zuvor stark gestiegenen Hormonspiegel gekennzeichnet. Im Durchschnitt 2 Tage nach der Geburt zeigten euthyme Mütter im Vergleich zu Frauen, die noch nie schwanger gewesen waren, eine signifikante Reduktion des GMV [10, 33] sowie eine reduzierte Netzwerkkonnektivität im Ruhezustand [29]. Von der Volumenreduktion sind besonders Hirnareale betroffen, die an der Regulation von Emotionen sowie sozialen und emotionalen Konflikten und Stress beteiligt sind [9, 38, 47]. Im selben postpartalen Zeitfenster traten depressionsähnliche Symptome auf, insbesondere Stimmungsschwankungen und Reizbarkeit, die als „Babyblues“ bezeichnet werden und bis zu 55–60 % aller neuen Mütter betreffen, wobei bis zu 10 % einen starken Babyblues erleben (Abb. 1; [11, 42, 43]).

In den ersten 6 Wochen postpartum – der subakuten Phase – normalisiert sich der Östradiolspiegel allmählich wieder, während der Progesteronspiegel jedoch erst langsam wieder ansteigt [5]. Die in der RiPoD-Studie erstmals angewendeten longitudinalen MRT-Messungen in 3‑wöchigen Abständen zeigten ebenfalls zeitspezifische Veränderungsmuster im Wochenbett, die mit den Progesteronspiegeln kovariierten [29, 33]. So finden in der subakuten postpartalen Phase die umfangreichsten und dynamischsten strukturellen sowie funktionellen Erholungsprozesse im Gehirn statt [29, 33], besonders in Gehirnregionen, die mit Stress- und Emotionsregulation assoziiert sind [4, 9] und eine Fülle von Steroidhormonrezeptoren aufweisen [3]. Basierend auf Analysen der Trajektorien der Hormonkonzentrationen im Wochenbett sowie der räumlichen Verteilung von Hormonrezeptoren im Gehirn vermuten wir, dass insbesondere der Progesteronspiegel diesen neuronalen Veränderungen zugrunde liegt [29, 33]. Dieses Zeitfenster birgt aber auch Gefahren für die psychische Gesundheit. So sind die ersten 4 bis 6 Wochen nach der Geburt der Zeitrahmen, der als postpartaler Beginn einer PPD definiert ist [1, 55]. Eine weitere postpartale Erkrankung, die innerhalb der ersten 4 Wochen nach der Geburt als Stressreaktion auftritt und ähnliche Symptome wie eine PPD aufweisen kann, ist die postpartale Anpassungsstörung (AS), die jedoch auf subklinischem Niveau verbleibt (Abb. 1; [55]).

## Prädiktoren für PPD auf der Grundlage postpartaler Nachuntersuchungen

In der RiPoD-Studie diagnostizierten wir am Ende des Beobachtungszeitraums anhand der Diagnosekriterien nach ICD-11 bei 9 % der Teilnehmerinnen eine PPD [22, 51], während die internationale Literatur Prävalenzen von 5–20 % in Industrieländern angibt [19, 28, 53]. Darüber hinaus fanden wir eine 17 %-Prävalenz der AS [22, 51], was in der überwältigenden Mehrheit der Literatur im internationalen Vergleich unberücksichtigt bleibt. Die Verwendung von Selbstberichtsskalen zu einem einzigen Zeitpunkt in der Wochenbettperiode birgt ein hohes Risiko, depressive Symptome, die sich noch im unterschwelligen Bereich (AS) befinden, als Symptome einer PPD fehlzuinterpretieren. Studien, die lediglich die Edinburgh Postnatal Depression Scale (EPDS; [13]) als Diagnoseinstrument verwenden, berichten von einer globalen PPD-Prävalenz von etwa 17 %, während Studien, die die DSM-Kriterien für die Diagnose verwenden von 10 % ausgehen [53], was sich mit unserer Prävalenz deckt.

Unter Umständen können die unterschiedlichen Prävalenzen auch daraus resultieren, dass es nicht immer leichtfällt, die Anzeichen einer depressiven Erkrankung zu erkennen und sie von anderen Stimmungsstörungen zu differenzieren. Die Möglichkeiten eines Screenings zur Identifikation, noch bevor ein Arzt oder Psychologe die Diagnose anhand der ICD-Kriterien stellen kann, ist gefragter denn je. So kann insbesondere die sogenannte digitale Phänotypisierung oder das Ecological Momentary Assessment (EMA) eine optimale Methode bieten, die psychische Gesundheit im Wochenbett zu beobachten und zu bewerten [37, 45]. Wenige Tage nach der Geburt werden Frauen häufig im euthymen Zustand nach Hause entlassen, wodurch ein etwas später erfolgender Abfall der Stimmung, sei es im Rahmen eines Babyblues, einer AS oder einer PPD, nur durch die Betroffene oder Angehörige erkannt werden kann. Mithilfe des EMA erfassten wir in der RiPoD-Studie in 3‑wöchigen Intervallen den Verlauf der selbstberichteten depressiven Symptome anhand des EPDS. Die nach 12 Wochen postpartal gestellten Diagnosen unterstützten die Annahme, dass die Mehrheit der Frauen mit einer PPD eine Verschlechterung der Symptome im Verlauf des Wochenbettes aufwies, während die Mehrheit der Frauen mit einer AS eine entsprechende Verbesserung [12] zeigte, wodurch eine Differenzierung der Störungen im Verlauf des Wochenbettes möglich wurde. Andere Studien, die ebenfalls die Stimmungstrajektorien im Wochenbett erfasst haben, zeigten ebenfalls eine Heterogenität in den Verläufen der depressiven Symptome, darunter erhöhte und sich verschlechternde Werte über das gesamte erste Jahr [8, 40], einen Höhepunkt an depressiven Symptomen in der 2. Woche nach der Geburt [38] und eine spontane Auflösung nach 7 bis 8 Wochen [8, 15, 38], wobei aufgrund der ausschließlichen Verwendung von Selbstberichtsfragebögen PPD und AS klinisch nicht unterschieden wurden. Dies wirft die Frage auf, ob die selbstheilenden Symptome kurze Episoden einer PPD darstellten oder ob es sich stattdessen um eine AS handelte, was das Haupt- und Alleinstellungsmerkmal unsere Arbeiten ist [12, 22]. An dieser Stelle sei erneut zu betonen, dass ein Remote-Screening zur möglichen Diagnosestellung höchst relevant ist. Bisher haben nur weniger Studien ein Screening des Stimmungsverlaufs im Wochenbett zur frühzeitigen Identifikation einer PPD verwendet. Dabei wurden jedoch häufig transiente depressive Symptome der AS und des Babyblues vernachlässigt [16, 17, 52], das PPD-Outcome nur anhand eines Screenings nach 4 Wochen erfasst, was ebenfalls zu einer Überlappung mit AS-Symptomen führte [52], oder depressive Symptome bereits am 2. Tag nach der Geburt gemessen, wodurch die Symptome des Babyblues oder einer bereits bestehenden Depression einbezogen wurden und zu unzureichenden Spezifitäten führten (47,69 %; [17]). In der RiPoD-Studie haben wir die PPD nach 12 Wochen anhand der ICD-11-Kriterien diagnostiziert und konnten rückwirkend unter Verwendung des EPDS direkt nach Geburt und 3 Wochen nach Geburt sowie einer 2‑tägigen Onlineabfrage der aktuellen Stimmung bereits in der 3. Woche nach Geburt mit einer 87 %igen Genauigkeit frühzeitig zwischen PPD und gesundbleibenden Frauen differenzieren [22]. In Woche 6 war eine Unterscheidung mit 76 %iger Genauigkeit zwischen AS und PPD möglich. Dieser Zeitpunkt stellt somit einen Wendepunkt im Wochenbett dar, ab dem AS und PPD phänotypisch auseinanderdriften und sich klar voneinander unterscheiden lassen. Mithilfe engmaschiger Onlinebefragungen lässt sich die PPD somit gut von reaktiven Symptomen abgrenzen, die möglicherweise eine Reaktion auf die Geburt und die damit verbundenen veränderten Lebensumstände darstellen können.

## Hormonelle Risikofaktoren einer PPD

Die Untersuchung jeder einzelnen postpartalen Frau, sei es remote oder in Person, wird in vielen Fällen aufgrund eingeschränkter Ressourcen nicht durchführbar sein. Daher ist es besonders wichtig, Frauen mit einem besonders hohen Risiko frühzeitig zu erkennen. Zu den allgemeinen Risikofaktoren, die mit einer PPD oder AS assoziiert sind, gehören eine persönliche Vorgeschichte mit depressiven Störungen, belastende Lebensereignisse, geburtsbedingte psychische Traumata und Komplikationen während der Geburt sowie eine familiäre Vorgeschichte mit psychiatrischen Störungen [20–22, 48, 56]. Jedoch scheinen insbesondere hormonell bedingte Veränderungen von größerer Relevanz zu sein [5]. Obwohl die Symptome des Babyblues spätestens nach 2 Wochen abklingen, bleiben sie bei einigen Frauen über längere Zeit bestehen und gehen in klinisch relevante Stimmungsstörungen über [11]. Im klinischen Kontext muss der Babyblues daher als wichtiger und spezifischer Risikofaktor für eine PPD angesehen werden [11, 23, 30, 42]. Neben dem Babyblues ist als weiterer spezifischer und hormonell bedingter Risikofaktor die Vorgeschichte eines prämenstruellen Syndroms (PMS) zu betonen [6, 7, 11, 48]. Die kontinuierliche Exposition gegenüber hohem Östradiol, Progesteron und seinem Metaboliten Allopregnanolon während der Schwangerschaft soll stimmungsstabilisierende Wirkungen haben [14, 36], während der Mangel an diesen Hormonen nach der Geburt mit Babyblues und PPD in Verbindung gebracht wird [18, 49]. Ähnlich erreichen die PMS-Stimmungssymptome ihren Höhepunkt in den letzten 5 Tagen vor der Menstruation, wenn der Progesteronspiegel zu sinken beginnt [34]. Babyblues, PMS und PPD können demnach als symptomatische (affektive) Reaktionen auf hormonelle Schwankungen betrachtet werden, wobei betroffene Frauen im Vergleich zu gesunden Frauen eine veränderte Empfindlichkeit gegenüber Pregnanolonmetaboliten (Allopregnanolon) zu entwickeln scheinen [52]. Neben der aktuellen pharmakologischen Anerkennung von Allopregnanolon und dessen Mangel als Quelle für Stimmungsstörungen [2, 14, 31, 49] und unserer beobachteten Kovarianz zwischen dem Verlauf des Progesteronspiegels und der postpartalen Neuroplastizität [29, 32] wird auch an dieser Stelle erneut deutlich, dass diese Neuroplastizität eng mit der Entwicklung postpartaler Stimmungsstörungen verbunden sein könnte.

## Vorhersage einer PPD: Ist es möglich und was wird dafür benötigt?

**Abb. 2:**
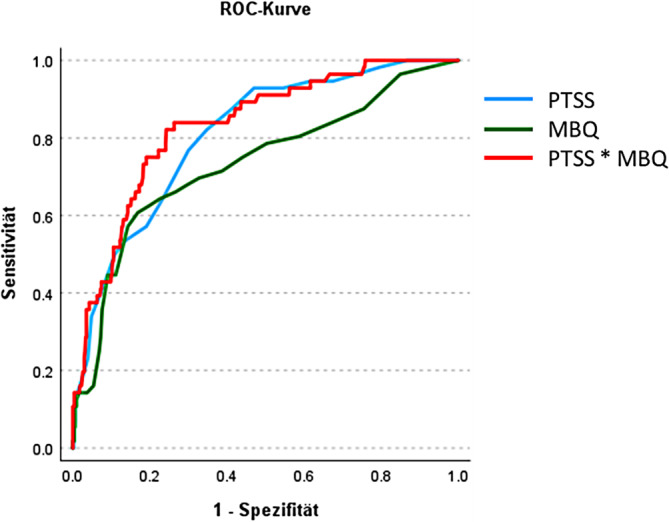
Receiver-Operating-Characteristic(*ROC*)-Kurve der Sensitivität vs. Spezifität der Vorhersage der postpartalen Depression basierend auf dem Maternity Blues Questionnaire (*MBQ*), der Premenstrual Tension Syndrome Scale (*PTSS*) und deren Kombination. *N* = 596 postpartale Frauen, von denen 9,4 % eine postpartale Depression entwickelten

Die spezifischen und hormonell bedingten Risikofaktoren erscheinen vielversprechend in der Identifizierung von Frauen mit hohem Risiko für eine PPD. So haben Frauen mit einem schweren Babyblues sowie Frauen mit einem schweren PMS jeweils ein 8‑ bis 10fach erhöhtes Risiko, im Laufe des Wochenbettes eine PPD zu entwickeln [11]. Die Kombination von schwerem Babyblues und schwerem PMS führt hingegen zu einem fast 13fach erhöhten Risiko für eine PPD. In einer zusätzlichen Untersuchung mit 596 postpartalen Frauen, von denen 9,4 % eine PPD und 20,3 % eine AS entwickelt haben, schätzten wir basierend auf den Screenings zu PMS-Symptomen (Premenstrual Tension Syndrome Scale, PTSS [46]) in Kombination mit einem Screening zu Babybluessymptomen (Maternity Blues Questionnaire, MBQ; [25]) mithilfe einer Receiver-Operating-Characteristic(ROC)-Analyse die optimalen Cut-off-Werte der Screenings zur Vorhersage der PPD. Mit einer Sensitivität von 82,1 % und einer Spezifität von 75,7 % (Abb. 2) können der PTSS und der MBQ jeweils mit einem Cut-off von 11 bzw. 12 eine PPD mit einer hohen Genauigkeit von 83,1 % vorhersagen.

## Empfehlungen für die Praxis

Um die PPD frühzeitig behandeln zu können, ist es von größter Relevanz, Frauen mit hohem Risiko zu identifizieren und eine PPD frühzeitig zu diagnostizieren. Das Screening von Frauen mit erhöhtem Depressionsrisiko kann optimiert werden, indem bereits in den ersten Tagen nach der Geburt, wenn sich die Frau möglichst noch in ärztlicher Obhut befindet oder durch Nachsorgehebammen betreut wird, sowohl der Schweregrad des Babyblues als auch die Vorgeschichte und der Schweregrad des PMS erfasst werden. Die Beurteilung des Babyblues und die Anamnese von PMS können dadurch zur frühzeitigen Identifizierung einer Hochrisikogruppe für die weitere Überwachung verwendet werden. Eine anschließende Beobachtung der Symptomentwicklung mittels einer digitalen Stimmungserfassung in den ersten 3 Wochen postpartal [22] kann helfen, sich entwickelnde depressive Symptome rechtzeitig zu erkennen. Die fragebogenbasierte Untersuchung direkt nach der Geburt sowie eine zusätzliche Fernbeurteilung 3 Wochen nach der Geburt können leicht in die Routineversorgung integriert werden. Dadurch können einerseits zwischen der transienten AS und der PPD unterschieden und andererseits die Dunkelziffer der nicht erkannten PPD-Fälle gesenkt werden. Beides würde eine frühe Anbindung der betroffenen Frauen an ein klinisch-therapeutisches Setting deutlich erleichtern.

## Schlussfolgerung und Perspektiven

Mithilfe der RiPoD-Studie konnten wir vertiefte Einblicke in die hochkomplexe postpartale Periode gewinnen, um das dynamische Zusammenspiel zwischen Hormonen, Neuroplastizität und postpartalen Störungen besser zu verstehen.

Weitere Längsschnittbeobachtungen sind jedoch notwendig, um die vielfältigen Prozesse in den emotionalen, physiologischen und neuroendokrinologischen Bereichen abzubilden. Dabei sollten solche Studien nicht nur punktuelle Veränderungen, sondern komplexe Trajektorien verschiedenster Bereiche (Stresswahrnehmung, Veränderung der Stimmung, hormonelle Anpassung, vorbestehende Vulnerabilität, neuronale Plastizität etc.) erfassen, um die Erstellung prädiktiver Modelle sowie ein besseres Verständnis von Ursachen und Folgen zu ermöglichen. So bleibt es beispielsweise noch unklar, inwieweit Hormonschwankungen, einschließlich der modulierenden Wirkung der neuroprotektiven Effekte von Östradiol und Progesteron, die Neuroplastizität beeinflussen und wie diese zur Entwicklung psychiatrischer Störungen beitragen. Es stellt sich auch die Frage, ob die normalen neuroplastischen Anpassungsprozesse bei gefährdeten oder an einer PPD erkrankten Frauen in ähnlicher Weise ablaufen oder ob es Hinweise darauf gibt, dass bereits diese Anpassungsprozesse beeinträchtigt sind. Ein besseres Verständnis dieser physiologischen Veränderungen bei gesunden Frauen könnte dazu beitragen, die Rolle der strukturellen Plastizität als Risikofaktor für die Entstehung postpartaler Stimmungsstörungen besser zu verstehen.

Des Weiteren sollte in diesem Zusammenhang der Begriff „Wochenbettperiode“ überdacht werden, da manche Frauen mit ähnlich langer Schwangerschaftsdauer einen medizinisch indizierten Schwangerschaftsabbruch oder eine Fehlgeburt erleben, jedoch nicht nur aus geburtshilflicher Sicht unterschiedlich behandelt. Dies geschieht trotz der Tatsache, dass PPD und Depressionen nach einem späten Schwangerschaftsabbruch denselben Ursprung haben können. Die Hormonspiegel sind bereits im 2. Trimester höher als im normalen Menstruationszyklus [5]. Daher ist auch ein Schwangerschaftsabbruch mit einer Reihe von Risikofaktoren für eine PPD verbunden, einschließlich den pränatalen Hormonfluktuationen. Zuletzt gewinnt auch die väterliche peripartale psychische Gesundheit zunehmend an Bedeutung. Da Depressionen bei Vätern nicht auf die bei Müttern beobachteten Veränderungen in endokrinen Systemregulierungen zurückzuführen sind, bietet die Forschung an Vätern eine einzigartige Gelegenheit, direkte geschlechtsspezifische Veränderungen im Verhalten, im Hormonsystem sowie in der Hirnstruktur und -funktion während des Übergangs zur Elternschaft zu untersuchen.

Zusammenfassend ist es wichtig, die physiologischen und biologischen Anpassungen, einschließlich ihrer Konsequenzen und Störungen, die während der gesamten Schwangerschaft auftreten, genauer zu untersuchen. Diese breitere Perspektive könnte unser Verständnis der neurobiologischen Auswirkungen von Schwangerschaft und ihrer Komplikationen verbessern und zu einer besseren Unterstützung und Intervention für alle Frauen führen.
